# Evolution of 2009 H1N1 influenza viruses during the pandemic correlates with increased viral pathogenicity and transmissibility in the ferret model

**DOI:** 10.1038/srep28583

**Published:** 2016-06-24

**Authors:** Anna Otte, Anthony C. Marriott, Carola Dreier, Brian Dove, Kyra Mooren, Thorsten R. Klingen, Martina Sauter, Katy-Anne Thompson, Allan Bennett, Karin Klingel, Debby van Riel, Alice C. McHardy, Miles W. Carroll, Gülsah Gabriel

**Affiliations:** 1 Viral Zoonoses and Adaptation, Heinrich Pette Institute, Leibniz Institute for Experimental Virology, Hamburg, Germany; 2 Public Health England, Porton Down, United Kingdom; 3 Department for Computational Biology of Infection Research, Helmholtz Center for Infection Research, Braunschweig, Germany; 4 Department for Molecular Pathology, Institute of Pathology, University Hospital Tübingen, Germany; 5 Erasmus Medical Center, Rotterdam, The Netherlands; 6 Center for Structure and Cell Biology in Medicine, University of Lübeck, Germany

## Abstract

There is increasing evidence that 2009 pandemic H1N1 influenza viruses have evolved after pandemic onset giving rise to severe epidemics in subsequent waves. However, it still remains unclear which viral determinants might have contributed to disease severity after pandemic initiation. Here, we show that distinct mutations in the 2009 pandemic H1N1 virus genome have occurred with increased frequency after pandemic declaration. Among those, a mutation in the viral hemagglutinin was identified that increases 2009 pandemic H1N1 virus binding to human-like α2,6-linked sialic acids. Moreover, these mutations conferred increased viral replication in the respiratory tract and elevated respiratory droplet transmission between ferrets. Thus, our data show that 2009 H1N1 influenza viruses have evolved after pandemic onset giving rise to novel virus variants that enhance viral replicative fitness and respiratory droplet transmission in a mammalian animal model. These findings might help to improve surveillance efforts to assess the pandemic risk by emerging influenza viruses.

The first pandemic of the 21^st^ century was caused by a triple reassortant influenza A virus of the H1N1 subtype containing avian, swine and human gene segments^1^,^2^. Retrospective estimations suggest that >280,000 people died already during the first year of the pandemic^3^. Moreover, there is increasing evidence that 2009 pandemic H1N1 (2009 pH1N1) influenza A viruses (IAV) have further evolved after pandemic declaration on the 11th June 2009 by the World Health Organization, which might have additionally contributed to disease severity in the human population^4^,^5^.

In the UK, three distinct influenza waves were reported with the third epidemic causing a greater burden of severe illness compared to the previous year^4^,^6^. Epidemiological analysis revealed that 2009 pH1N1 IAV must have evolved and increased their fitness in the human host by the end of 2010^4^. This third epidemic in winter 2010/2011 was unexpected given the low susceptibility of the human population after two subsequent infection waves. Thus, it was proposed that increased viral transmissibility was the major contributor to increased viral spread and disease severity observed in humans^4^.

To date, many molecular studies have been performed identifying human-adaptive signatures in viral genes that increase viral replicative fitness in cell culture experiments, virulence in the murine host or transmissibility in ferrets compared to previously circulating seasonal IAV^5^,^7^,^8^,^9^,^10^,^11^. However, it remained unclear which viral mutations were introduced after pandemic declaration that might have contributed to elevated viral transmission and illness among the human population.

Therefore, in this study, we sought to analyse viral amino-acid positions that notably increased their frequency after pandemic onset and might have contributed to differential virus transmission and disease severity using two human isolates representative for early and late pandemic phases, respectively, in the ferret model.

## Results

### Increased frequency of specific 2009 pH1N1 amino-acids after pandemic declaration

**Figure 1:**
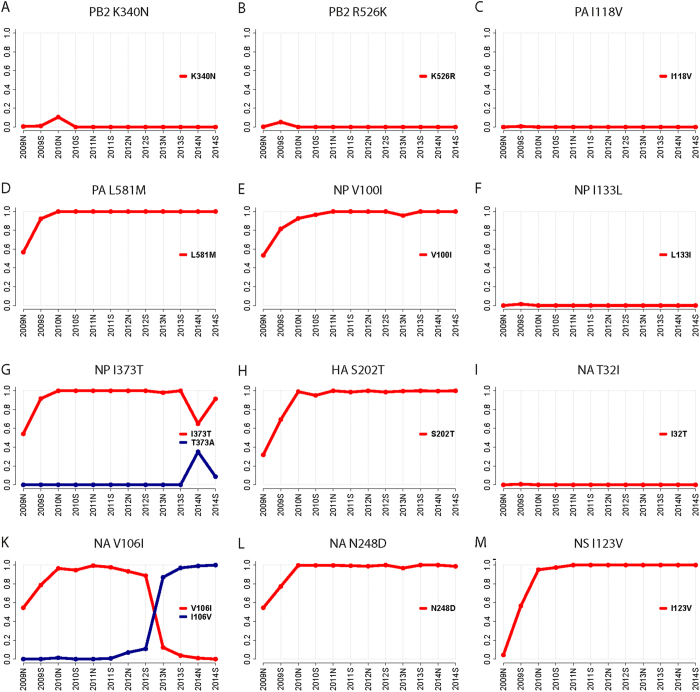
HH15-specific allele frequencies of 2009 pH1N1 strains isolated from 2009 to 2014. Allele dynamic (AD) plots were constructed based on phylogenetic viral data. The figure shows the evolutionary dynamics of positions differing from HH05 in the coding sequences of PB2 (**A**,**B**), PA (**C**,**D**), NP (**E**–**G**), HA (**H**), NA (**I**–**L**) and NS (**M**). Each plot shows the seasons (from 2009S to 2014S) on the x-axis and the allele frequency on the y-axis. Individual plots illustrate the changes in frequency of alleles that surpasses a frequency of at least 30% over consecutive seasons. The allele to each curve is colour coded and depicted in a separate legend within each plot. For the HA protein the H1 numbering was applied^30^.

First, we have analysed individual frequencies of viral amino-acid positions that were present before, during and five years after the pandemic declaration in June 2009. Therefore, we searched for amino-acid frequencies of 2009 pH1N1 isolates representative of the early (HH05 virus; isolated in April) or the late (HH15 virus; isolated in June) pandemic phase with particular emphasis on those amino-acid substitutions that emerged after pandemic declaration^5^. In general, the highest frequency was observed for 6 out of the 12 HH05/HH15-specific mutations that emerged after pandemic declaration in the northern as well as the southern hemisphere (PA-L581M, NP-V100I, NP-I373T, HA-S202T, NA-N248D and NS1-I123V) (Fig. 1). From these 6 mutations the early phase HH05-specific amino-acid positions were highly prevalent before the initiation of the pandemic (2009 northern hemisphere (N): 1^st^ October 2008–31^st^ March 2009) indicated by high amino acid frequencies: 100% for PA-L581, NP-V100, NP-I373, HA-S202, NS1-I103 and 50% for NA-N248 (Supplementary Fig. 1). Later, these positions lost dominance to the late phase HH15-specific amino-acids (2009 southern hemisphere (S): 1^st^ April 2009–30^th^ September 2009). In contrast, the other HH05/HH15 mutations, PB2-K340N, PB2-R526K, PA-I118V, NP-I133L, NA-T32I and NA-V106I either did not emerge to high frequencies or even back mutated from HH15-specific to HH05-specific amino-acids at later stages of the pandemic. Today, more than 99% of all circulating 2009 pH1N1 strains contain HH15-specific amino-acids at these indicated positions that emerged after pandemic declaration (Fig. 1D,E,G,H,L,M; Supplementary Fig. 1)^5^.

The bioinformatic analysis clearly shows that certain 2009 pH1N1 amino-acids emerged after pandemic declaration and represent the majority of the 2009 pH1N1 IAV that circulate today. Moreover, these identified high-frequency amino-acids might confer viral fitness and thus explain their dominant prevalence among the human population.

### HH15-specific amino-acids do not alter viral attachment to the respiratory tract

Increased viral respiratory droplet transmissibility is considered to be prerequisite for pandemic spread. Herein, particularly viral attachment to cells of the upper respiratory tract (URT) mediated by the viral hemagglutinin (HA) seems to be particularly important to ensure viral transmission from human-to-human^12^,^13^. Therefore, we first analysed the receptor binding of the differential pandemic phase viruses to cells of the upper and lower respiratory tract (LRT) of ferrets as an established animal model to mimic IAV transmission in humans.

**Figure 2:**
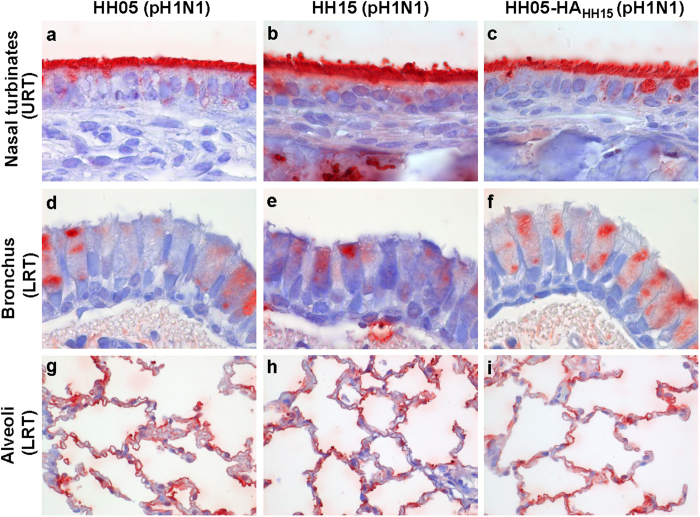
HH05 and HH15 viral attachment to the respiratory tract of ferrets. Tissue sections from the nasal turbinates (**a**–**c**) (designated as upper respiratory tract; URT), bronchi (**d**–**f**) and alveolar tissue (**g**–**i**) (designated as lower respiratory tract; LRT) of ferrets were used for virus attachment. Recombinant 2009 pH1N1 viruses HH05 and HH15 as well as single HA gene reassortant virus (HH05-HA_HH15_; C,F,I) were FITC-labelled and incubated with tissue sections. Virus attachment was detected by secondary HRP-staining in red. Tissues were counterstained with hematoxylin.

Both, HH05 and HH15 viruses attached to the URT as well as the LRT (Fig. 2). Inclusion of the single HH15-HA (hemagglutinin) gene reassortant HH05-HA_HH15_ virus did not alter viral tropism. All viruses efficiently attached to the apical side of ciliated epithelial cells in the URT. In the trachea and bronchi, viruses predominantly attached to goblet cells. In the bronchiole, to club cells and in the alveoli, predominantly to type I pneumocytes. Overall, HH05 as well as HH15 viruses both abundantly attached to the URT and the LRT of ferrets.

### HA S202T mutation increases virus binding to human-like α2,6-linked sialic acids

Viral attachment studies reveal alterations in recognition of viral receptors that are generally expressed on epithelial cells of the RT. However, they do not show potential differences in binding to distinct molecules abundantly expressed in the human RT, such as α2,6- or α2,3-linked SA^14^,^15^.

Therefore, we have analysed the SA binding properties of these viruses with modified red blood cells (RBC). Both, HH05 and the single gene reassortant HH05-HA_HH15_ virus containing HA-S202T did not bind α2,3-linked RBC (Table 1). However, the HH05-HA_HH15_ virus bound most efficiently to α2,6-linked RBC with 128 hemagglutination units (HAU) compared to HH05 with only 16 HAU. As controls, we used a human H3 and an avian H5 recombinant virus. The human H3 control virus bound to human-like α2,6-linked RBC with a high titre of 256 HAU, while binding to α2,3-linked RBC was only marginally detected with 2 HAU. As expected, the avian H5 control virus hemagglutinated α2,3-linked RBC to 512 HAU, while binding to α2,6-linked RBC was low with 8 HAU. These findings suggest that HA-S202T increases binding to α2,6-linked SA that are most abundantly expressed in the human URT.

### HH15-specific amino-acids confer increased viral replication in the respiratory tract of ferrets

For the establishment of a productive infection, viruses need to replicate in the respective host cells after receptor binding.

**Figure 3:**
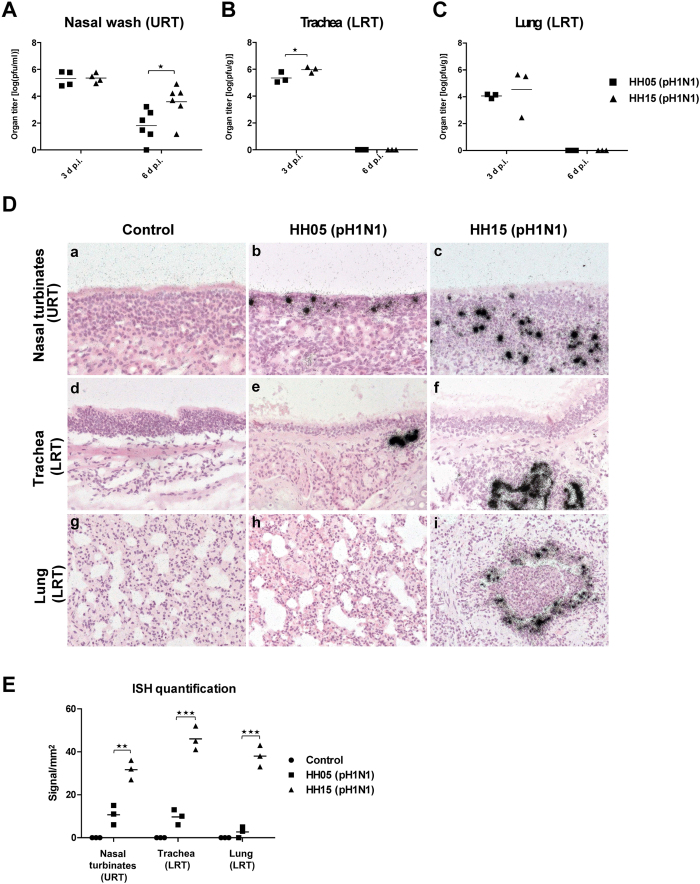
Replicative fitness of HH05 and HH15 in the respiratory tract of ferrets. Ferrets were intranasally infected with 10^5 ^p.f.u. of HH05 (pH1N1) (*n* = 6) or HH15 (pH1N1) (*n* = 6) viruses. On days 3 and 6 p.i., viral titres were determined in the nasal washes (*n* = 3–6) (designated as upper respiratory tract; URT) of infected animals by plaque assay (**A**) or animals were euthanized (*n* = 3) and viral titres determined using homogenates of trachea (**B**) and lung (**C**) (designated as lower respiratory tract; LRT) on days 3 and 6 p.i. For detection of viral RNA by *in situ* hybridization, nasal turbinates (URT), trachea (LRT) or lungs (LRT) were removed on day 3 p.i. (**D**). For quantification of ISH signals virus RNA-positive cells per mm^2^ were counted on 3 arrays per animal (**E**). Organs from animals treated with PBS only served as a control. Statistical analysis was performed using student’s *t*-test (asterisk, *p* ≤ 0.05; two asterisks, *p* ≤ 0.01; three asterisks, *p* ≤ 0.001).

HH15 replicates to elevated virus titres in the RT of ferrets, predominantly in the nasal turbinates compared to HH05-infected animals (Fig. 3A–C). The enhanced replicative fitness in HH15 infected ferrets correlated with an increased number of viral RNA-positive cells, in the URT (Fig. 3D(c)) as well as the LRT (Fig. 3D(f,i)). Compared to HH15, HH05 infected animals displayed only a few viral-RNA positive cells in the olfactory epithelium of the nasal turbinates (Fig. 3D(b)) and the bronchial epithelium (Fig. 3D(e)), while no or only single virus signals could be detected in the alveolar epithelium (Fig. 3D(h)). Moreover, HH15-infected ferrets showed severe inflammation, especially in the LRT (Fig. 3D(f,i)) in contrast to HH05 infected groups where little or no signs of inflammation was detected (Fig. 3D(b,e,h)) similar to that observed among uninfected control groups (Fig. 3D(a,d,g)). However, none of the infected ferrets displayed a significant weight loss or changes in body temperature (Supplementary Fig. 2) suggesting a general mild infection course upon both IAV infections. Thus, HH15-specific amino-acids confer an increased viral replicative fitness in the mammalian RT.

### HH15-specific amino-acids mediate elevated transmissibility via respiratory droplets in ferrets

Next, we addressed the question whether the increased HH15-HA binding to α2,6-linked SA as well as the generally increased replicative fitness of HH15 in the RT might affect transmissibility between mammals as a prerequisite of efficient pandemic spread.

**Figure 4:**
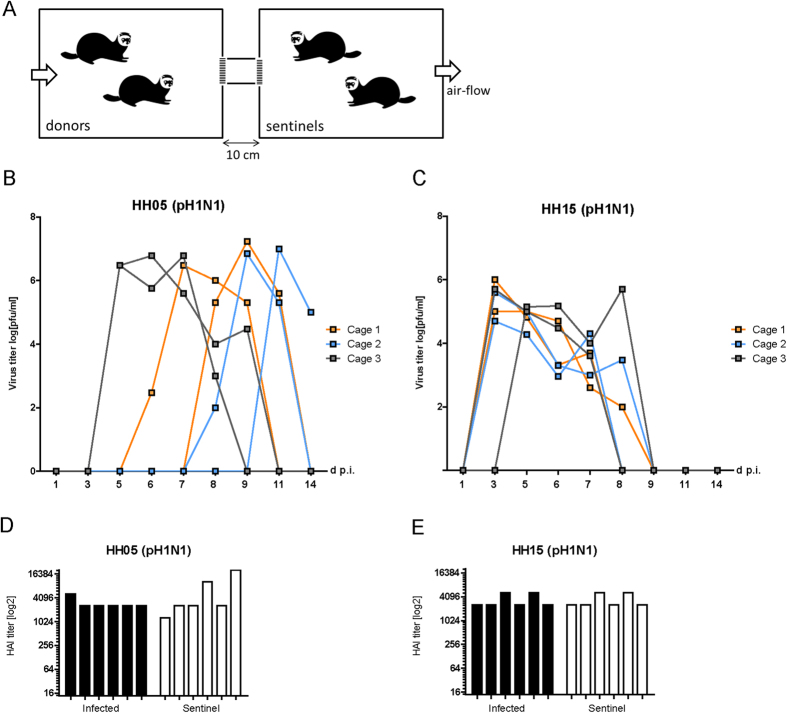
Respiratory droplet transmissibility of HH05 and HH15 between ferrets. (**A**) Donor ferrets were experimentally infected and sentinels introduced into the downstream cages 24 hr later. A 10 cm tunnel with metal mesh at each end separated the cages, allowing the passage of aerosol and small respiratory droplets. (Ferret icon designed by Dan Stack from The Noun Project). Ferrets were intranasally infected with 10^5 ^p.f.u. of HH05 (pH1N1) (*n* = 6) (**B**) or HH15 (pH1N1) (*n* = 6) (**C**) viruses. Cages containing sentinel animals (*n* = 6) were placed next to infected cages for 14 days. Viral titres were determined in the nasal washes of sentinel animals on days 1–14 p.i. by plaque assay. Hemagglutination inhibition (HI) titres were determined in the sera of infected and sentinel animals on days 14 or 21 p.i., respectively (**D**,**E**). Seroconversion was determined by means of HI titres above background levels of uninfected control groups.

Therefore, we infected donor animals and placed uninfected sentinels into adjacent cages (Fig. 4A). Both sentinel groups that were either exposed to HH05 or HH15 infected donor animals showed virus titres in their nasal washes suggesting that both viruses transmit via respiratory droplets (Fig. 4B,C). This was further supported by the hemagglutination inhibition (HI) titres against the respective virus strains in both sentinel groups exposed to HH05 or HH15 IAV infection (Fig. 4D,E).

However, some key transmission parameters, such as peak virus titres in donor animals, the time point of first viral detection as well as the transmission pattern were considerably different between HH05 and HH15 sentinels (Table 2). Interestingly, sentinel ferrets exposed to HH05 infected animals showed high virus titres (6.9 log p.f.u./ml) compared to sentinels exposed to HH15 infected animals (5.5 log p.f.u./ml). Moreover, initial virus titres were detected on 7.2 days post exposure to HH05 in contrast to those exposed to HH15 infected animals where viral titres were detected in average as early as 3.3 days post exposure. Furthermore, the delay in detection of initial virus titres in the URT of the second co-caged sentinel was 1.7 days in average. Indeed, taking the cage setting into account, one might speculate that up to half of the HH05 infected groups were infected via direct contact rather than respiratory droplet transmission (Fig. 4). There, two sentinel animals placed in the same cage mostly showed delayed virus titres in contrast to HH15 infected sentinels where five of six animals presented detectable virus titres in parallel supportive of respiratory droplet transmission.

Thus, both HH05 and HH15 viruses transmit efficiently via respiratory droplets to exposed sentinels in the ferret transmission model. However, HH15 shows an improved transmission pattern compared to HH05.

## Discussion

The 2009 pH1N1 IAV have evolved after pandemic onset resulting in viral variants that still circulate among the human population^4^,^5^,^10^. However, it remained unclear whether these genomic alterations affect viral fitness among humans. To study this question, we have used the 2009 pH1N1 strain named HH05 that belongs to the clade 1 lineage as one of the earliest and most homogenous clades that emerged in the pandemic and probably circulated for approximately 2 to 6 weeks before its initial detection. We compared the amino-acid frequencies of HH05 to the 2009 pH1N1 strain named HH15 that belongs to the previously reported clade 7, which is representative of the 2009 pH1N1 strains that circulated later during the pandemic after April 2009^5^,^16^. We could identify 6 mutations in the HH15 viral genome (PA-L581M, NP-V100I, NP-I373T, HA-S202T, NA-N248D and NS1-I123V) that became prevalent after pandemic declaration with high frequency. Today, these positions represent more than 99% of currently circulating H1N1 IAV suggestive of their selective advantage among the human population.

Modelling studies revealed that increased transmissibility of 2009 pH1N1 viruses acquired after pandemic onset was the major contributor to severe disease outcome during the third wave in winter 2010/2011 in the UK^4^. HH15-specific sites in NP-100I, HA-202T and NS1-123V were highly prevalent in 2009 pH1N1 strains isolated during the 3rd and most severe wave (2010 to 2011) of the pandemic with increased hospitalizations and deaths compared to the first two waves in the UK^10^. Since the HH05-specific sites have almost disappeared among circulating strains in the post-pandemic phase, we proposed that HH15-specific sites might represent human-adaptive mutations that confer viral fitness and pandemic spread.

Here, we show that the HH15 strain displays a more efficient transmissibility via respiratory droplets compared to HH05 in ferrets. This correlates with increased HH15 virus replication in the ferret RT. However, HH05 virus titres were higher in the URT in the sentinel animals after virus transmission. Thus, a high virus titre in the URT is neither sufficient nor does directly correlate with increased viral transmissibility in line with previous reports^17^. HH15 was not more lethal compared to HH05 in the ferret model. However, lungs of HH15-infected ferrets presented substantial inflammation as detected by infiltrating mononuclear cells. Since the 2009 H1N1 influenza pandemic has caused a substantial burden in patients with underlying co-morbidities^3^, the ability of HH15 to replicate to increased virus titres in the RT as well as to cause inflammation might have contributed to severe disease among these high-risk groups. In the mouse model, we showed earlier that HH15 is more virulent than HH05^5^,^18^,^19^. There, HH15-specific mutations in HA-S202T, NP-V100I, NP-I133L and NP-I373T were responsible for increased virulence. Herein, mutations in NP elevated viral replication in human and murine lung cells and mediated lymphopenia^5^. Thus, HH15-specific mutations cause severe lung pathology in the immunocompetent mouse and ferret models. However, it should be noted that despite having used ferrets as the gold standard to model human influenza, these are still animal and not human data which should be considered as a limitation of this study.

Regarding the molecular basis of increased transmissibility via respiratory droplets, we identified the HA-S202T mutation present in the HH15 strain as a key site that increases 2009 pH1N1 influenza binding to human-like α2,6-linked SA, which are most abundantly expressed in the human URT^14^,^15^ and are crucial for viral respiratory transmissibility^12^,^13^. The HA-202 amino-acid position is located in the globular head domain opposite of the receptor binding site of HA. However, it is known that mutations in the globular head might indirectly influence its receptor binding, even if the receptor binding pocket is not directly affected^20^. Consistently, it was reported that in the first years of 2009 pH1N1 evolution, mutations in HA that increase HA receptor-binding avidity, including the HA 202 position but only in combination with other amino acid positions, were followed by the selection of compensatory mutations that restore binding to the original levels^21^. However, the HA-S202T substitution did not affect viral features often associated with differential transmissibility, such as impaired antigen recognition (Supplementary Fig. 3) or virus stability (Supplementary Fig. 4). Thus, viral replicative fitness in the RT as well as enhanced binding to receptors most abundant in the human URT are direct correlates of more efficient transmissibility via respiratory droplets in the ferret model. Further studies are required to assess whether a single mutation in HA is sufficient to confer more efficient respiratory droplet transmission or whether the effects are only observed when all HH15-mutations are combined.

Other mutations in PB2 or HA might also affect transmissibility of 2009 pH1N1 viruses in ferrets. Mutations in PB2-A271T and HA-Q226R abolished respiratory droplet transmission in ferrets^11^. Similarly, the PB2-R591Q mutation reduced 2009 pH1N1 virus respiratory droplet transmission between ferrets^22^. However, HH05 and HH15, both contain PB2-271A and PB2-591R as well as HA-226Q (H3 numbering). This suggests that while these positions might have contributed to initial viral spread, 2009 pH1N1 viruses have acquired additional mutations in HA (such as HA-S202T) within the first months after pandemic declaration in June 2009 that further increases transmissibility via respiratory droplets and warrants subsequent spread among the human population. This hypothesis is further supported by the fact that >99% of currently circulating H1N1 strains harbour these HH15-specific positions. However, we cannot exclude that additional adaptive mutations have occurred that might also affect viral transmissibility but have not been investigated in this study.

In summary, our findings here suggest that increased vigilance in viral surveillance is required even after pandemic onset since IAV seem to harbour the potential to further evolve causing severe subsequent epidemics in the human population.

## Materials and Methods

### Cells and viruses

MDCK (Madin-Darby canine kidney) cells were grown in MEM (minimal essential medium; PAA) supplemented with 10% FCS (fetal calf serum; PAA), 1% glutamine (PAA) and 1% penicillin/streptomycin (PAA).

The 2009 pH1N1 wild type viruses A/Hamburg/05/09 (abbreviated as HH05) and A/Hamburg/NY1580/09 (abbreviated as HH15) were isolated on the 28^th^ April and 7^th^ June 2009, respectively, from pharyngeal swabs of patients before oseltamivir treatment as described before^19^. Recombinant 2009 pH1N1 viruses were generated by reverse genetics using the pHW2000 based 8-plasmid system^5^. All viruses, including the recombinant A/Netherlands/213/03 (H3N2) and A/Vietnam/11/94 (H5N1) strains were propagated on MDCK cells.

### Prevalence and allele dynamics of HH15-specific signatures

Coding sequences and amino-acid sequences from 2009 pH1N1 IAV with isolation dates from 2009 to 2014 were downloaded for the proteins PB2, PA, NP, HA, NA and NS1 from the EPIFLU Database^23^ (Number of isolates per protein: PB2 3725, PA 3453, NP 3501, HA 11837, NA 10392, NS1 4064). For each protein, the same analysis framework was applied:

Duplicates and isolates without a specified isolation month were removed to allow a precise assignment to the corresponding season. Following the standard definitions, the Northern hemisphere season begins on 1^st^ October and ends on 31^st^ March in the following year whereas the Southern hemisphere season begins on 1^st^ April and ends on 30^th^ September in the same year. Further, we sampled 300 isolates per season and guaranteed that our data comprises a nucleotide and an amino-acid sequence for each isolate. Nucleotide and amino-acid sequences were aligned with Muscle^24^ and automatically curated with trimAl^25^ to improve the alignments.

Based on the sequence alignments, a phylogenetic tree with approximately-maximum-likelihood inference was constructed using FastTree (version 2.1.7)^26^. We defined the sequence A/California/05/2009 (EPI176493) as an outgroup and inferred character state reconstruction of amino-acid intermediates. The genealogy was used to calculate the frequencies of alleles and corresponding amino-acid substitutions. Frequencies were subsequently plotted over consecutive seasons in allele-dynamics (AD) plots^27^ to indicate alleles changing most rapidly in frequency. Alleles likely being subject to directional selection can be determined as increasing in frequency and rising to fixation in the population^25^.

Based on the HA alignment an approximately-maximum-likelihood phylogenetic tree was calculated with fastree (version 2.1.7)^26^ and an allele-dynamics (AD) plot^27^, calculated from the data to determine alleles and corresponding amino-acid exchanges increasing most rapidly in frequency over consecutive seasons. The sequence A/California/05/2009 (EPI176493) was used to root the tree^27^.

### Virus attachment to respiratory tract tissues of ferrets

Recombinant 2009 pH1N1 viruses were purified with PEG (BioVision) according to the manufacturer’s protocols. For deactivation of viruses, dialysis was performed against 0.1% Formalin. Viruses were labelled with FITC (Sigma) as described before^15^. Briefly, concentrated virus was diluted 1:1 with 0.1 mg/ml FITC solution and incubated 1 h at room temperature. Tissue sections from the respiratory tract (RT; including the nasal turbinates, trachea, bronchi, bronchiole and alveoli) of ferrets (*n* = 3) were deparaffinised and stained with FITC-labelled viruses. After overnight incubation with FITC-labelled viruses, FITC was detected using a rabbit anti-FITC-HRP (DAKO). Additionally, a Tyramide Signal Amplification kit (Perkin Elmer) was used. Peroxidase was revealed using AEC and tissues were counterstained with hematoxylin.

### Receptor binding to erythrocytes

The hemagglutination (HA) assay was performed to assess the receptor binding specificity^28^. Briefly, sialic acids (SA) were removed from the surface of turkey red blood cells (RBC) in PBS by incubation with 50 mU *Vibrio cholerae* NA (VCNA; Roche) in 8 mM calcium chloride at 37 °C for 1 hour. Resialylation was performed using 0.5 mU of α2,3-(N)-sialyltransferase (Calbiochem) or 2 mU of α2,6-(N)-sialyltransferase (Japan tobacco) and 1.5 mM CMP-sialic acid (Sigma-Aldrich) at 37 °C for 2 h to produce α2,3-RBC and α2,6-RBC, respectively. After washing, the RBC were resuspended in PBS containing 1% bovine serum albumin to a final concentration of 0.5% RBC.

### Ethics statement

The ferret experiments were performed according of the United Kingdom Home Office Animals (Scientific Procedures) Act 1986. All animal protocols were approved by the Animal Welfare and Ethical Review Body of Public Health England (Porton), as required by the UK Home Office Animals (Scientific Procedures) Act, 1986 and performed in the animal facility of Public Health England, Porton Down, United Kingdom. Ferret tissues for *ex vivo* receptor binding studies as well as turkey red blood cells were obtained from the Erasmus Medical Center, Rotterdam, Netherlands, according to the guidelines of the Dutch animal protection law in accordance with approved protocols by the relevant Dutch authorities.

### Animal experiments

Ferrets were purchased from the Highgate Farm facility, United Kingdom. 6–12 months old female ferrets were anesthetized intra-muscularly with ketamine/xylazine (18 mg/kg; 1.4 mg/kg) and inoculated intranasally with 200 μl inoculums containing 10^5 ^p.f.u. of 2009 pH1N1 virus diluted in PBS. Control groups or sentinel animals received PBS only. Ferrets were observed for 14 days for survival, weight loss and body temperature. On several days p.i., all animals were narcotized and nasal wash was performed with 1 ml PBS (+0,2% BSA; +1% Pen/Strep). On days 3 and 6 p.i., three animals were euthanized and trachea and lungs were removed and homogenized. Virus titres were determined by plaque assay on MDCK cells as described before^19^.

### *In situ* hybridization

Virus RNA in tissues was detected using single-stranded ^35^S-labelled viral RNA probes, which were synthesized from a pBluescript II KS+ vector containing a fragment of the NP gene (nt 1,077–1,442) of A/Hamburg/NY1580/09 (pH1N1) as reported before^19^. Finally, slides were subjected to autoradiography, exposed for 3 weeks at 4 °C and counterstained with eosin^19^.

### Transmission experiments

For respiratory droplet transmission experiments, 3x two ferrets were infected with 10^5 ^p.f.u. (200 μl inoculums) of the 2009 pH1N1 viruses in a cage separated by 10 cm distance to a second cage where 3x two sentinel animals were placed on day 1 p.i. similar to previously reported settings^12^,^13^. Air was drawn through the cages at a rate of 20 changes per hour. Exhaust air was passed through a HEPA filter. Weight loss and survival was observed of all animals for 14 days p.i. and virus titres were determined in nasal washes at multiple time points p.i.

### Hemagglutination inhibition assay

Seroconversion of sentinels was analysed by hemagglutination inhibition (HI) of heat-inactivated serum samples (56 °C, 30 min) of the animals using 1% chicken erythrocytes that were purchased from Lohmann Tierzucht, Cuxhaven, Germany. HI titres were read as the maximum dilution of serum where hemagglutination was inhibited.

### Aerosol stability of virus particles

Stability of 2009 pH1N1 virus particles was determined with the Goldberg drum methodology as described before^17^,^29^. Air running though the Goldberg drum was allowed to equilibrate to 55% relative humidity for 30 min. One minute samples of aerosolized virus were collected at various time points thereafter (t = 0, 5, 15, 30, 45, and 60 min) and titrated by plaque assay.

## Additional Information

**How to cite this article**: Otte, A. *et al*. Evolution of 2009 H1N1 influenza viruses during the pandemic correlates with increased viral pathogenicity and transmissibility in the ferret model. *Sci. Rep*. **6**, 28583; doi: 10.1038/srep28583 (2016).

## Supplementary Material

Supplementary Information

## Figures and Tables

**Table 1:** HH15-HA sialic acid binding properties†.

Virus	Untreated RBC	VCNA RBC‡	α-2,3 SA RBC#	α-2,6 SA RBC#
HH05 (pH1N1)	64	0	0	16
HH05-HA_HH15_ (pH1N1)	64	0	0	128
H3 control	128	0	2	256
H5 control	128	0	512	8

^†^HA assays were performed with two fold dilutions of red blood cells (RBC) from turkey with recombinant HH05 virus and the single gene reassortant HH05-HA_HH15_ with HH15-specific HA in the HH05-background. As control viruses A/Netherlands/213/03 (H3N2; H3 control) and a 7 + 1 reassortant virus of A/PR/8/34 (H1N1) and the HA of A/Vietnam/11/94 (H5N1) without the basic cleavage site (H5 control) were used. Results from one representative run are shown in hemagglutination units (HAU) for each tested virus.

^‡^Removal of sialic acids (SA) was controlled after *Vibrio cholerae* NA (VCNA) treatment.

^#^Subsequently, RBC were either resialylated with α2,3-(N)-sialyltransferase or α2,6-(N)-sialyltransferase, respectively.

**Table 2:** Comparison of respiratory droplet transmissibility in sentinel ferrets exposed to HH05 or HH15 influenza virus infection†.

Virus	Peak virus titre‡ log[pfu/ml]	Day first titre [d post exposure]	Δ first titre cage# [days]
HH05 (pH1N1)	6.9 ± 0.2	7.2 ± 2.1	1.7 ± 1.5
HH15 (pH1N1)	5.5 ± 0.5***	3.3 ± 0.8**	0.7 ± 1.2

^†^Cages with sentinel ferrets (*n* = 6) were placed next to cages with pH1N1-infected ferrets (*n* = 6; 10^5 ^p.f.u.; 10 cm distance) for 14 days.

^‡^Viral titres were determined in the nasal washes of sentinel animals on days 1–14 p.i. by plaque assay. Sentinel animals were placed pair wise in cages.

^#^Delay of first virus titre in nasal wash of second sentinel animal per cage was calculated. Statistical analysis was performed using student’s *t*-test (two asterisks, *p* ≤ 0.01; three asterisks, *p* ≤ 0.001).
