# Supplementary material for: Evolution of 2009 H1N1 influenza viruses during the pandemic correlates with increased viral pathogenicity and transmissibility in the ferret model
Source: Sci Rep. 2016 Jun 24;6:28583. doi: 10.1038/srep28583 (PMC4919623; doi:10.1038/srep28583)
Supplement: Supplementary Information [file srep28583-s1.pdf]

Supplementary Information:

Evolution of 2009 H1N1 influenza viruses during the pandemic correlates with increased viral pathogenicity and transmissibility in the ferret model

Anna Otte, Anthony C. Marriott, Carola Dreier, Brian Dove, Kyra Mooren, Thorsten R. Klingen, Martina Sauter, Katy-Anne Thompson, Allan Bennett, Karin Klingel, Debby van Riel, Alice C. McHardy, Miles W. Carroll, Gülsah Gabriel

Supplementary figure legends

Supplementary Figure 1

HH15-specific amino-acid frequencies in 2009 pH1N1 viruses.

Sequences from 2009 pH1N1 strains isolated during the 2009 until the 2014 influenza season were downloaded from the EPIFLU Database for the northern hemisphere season (N) and the Southern hemisphere season (S). For indicated positions, amino-acid occurrences were calculated as frequencies relative to the total number of all sequences per season. HH05-specific amino-acids are indicated in green, while HH15-specific amino-acids are indicated in red. All other amino-acids are presented as shades of grey. For the HA protein the H1 numbering was applied [29].

Supplementary Figure 2

Weight loss and body temperature in HH05- and HH15-infected ferrets.

Ferrets were intranasally infected with 10^5 p.f.u. of HH05 (pH1N1) (*n*=9) or HH15 (pH1N1) (*n*=9) viruses. Controls received PBS only. Mean weight loss and standard deviations thereof are shown (A). Initial body weight was set 100%. Body temperature was determined twice per day. Here, the daily mean and standard deviations thereof are shown (B).

Supplementary Figure 3

Cross-reactivity of ferret sera against HH05 and HH15.

Ferrets were intranasally infected with 10^5 p.f.u. of HH05 (*n*=6) or HH15 (*n*=6) viruses (pH1N1). Hemagglutination inhibition (HI) titres against both 2009 pH1N1 virus isolates were determined in the sera of infected animals taken on days 14 p.i..

Supplementary Figure 4

Stability of HH05 and HH15 virus particles.

To determine virus particle stability, HH05 or HH15 (pH1N1) suspension was sprayed into a Goldberg drum for 10 minutes. One minute samples of aerosolized virus were collected at t = 0, 5, 15, 30, 45, and 60 min. The ratio of virus to the theoretical dilution was determined for each sample, averaged for duplicates and expressed as a percentage of the baseline (t = 0) ratio. Experiment was repeated in triplicate for each virus strain.

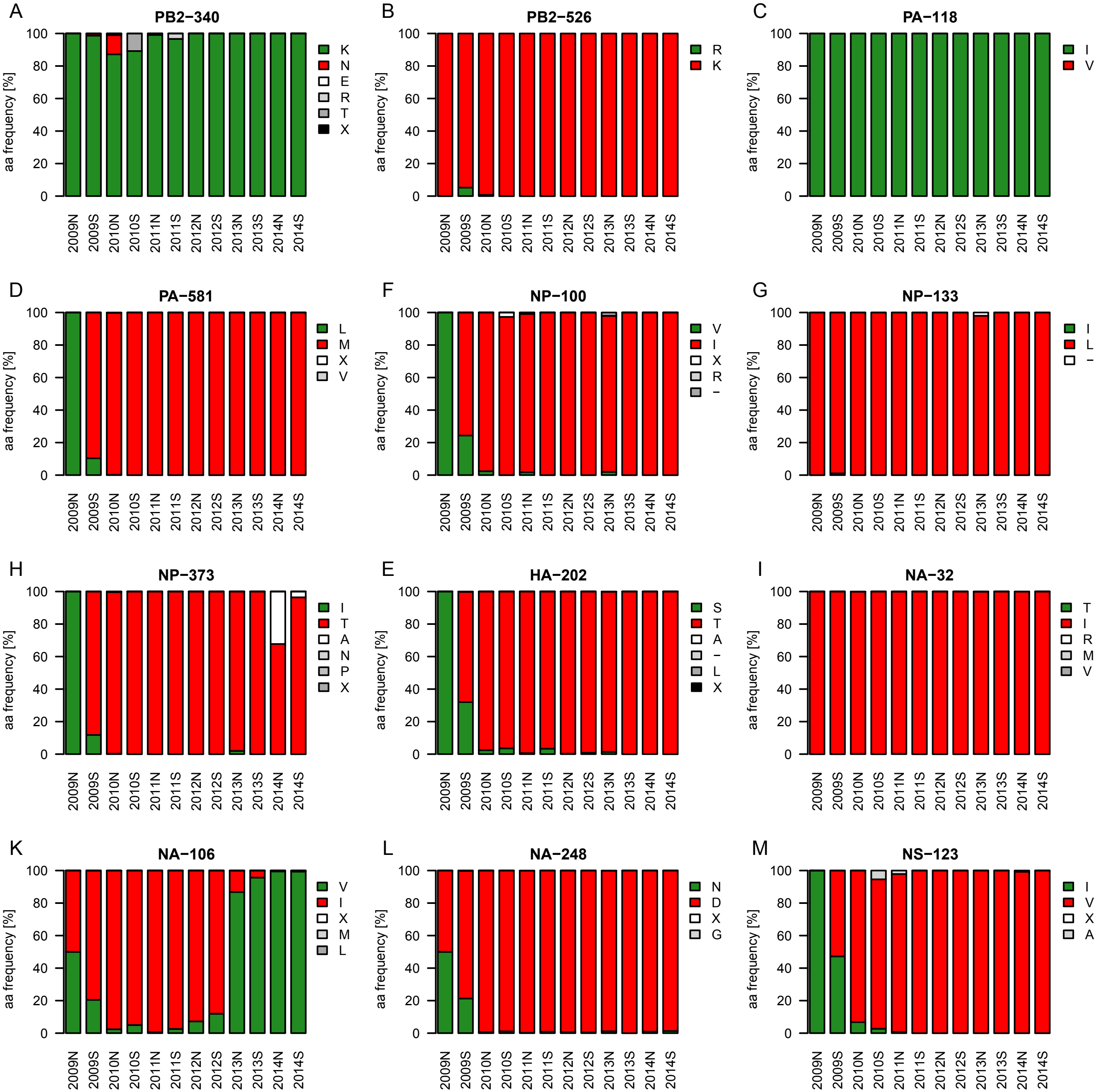

Supplementary Figure 1

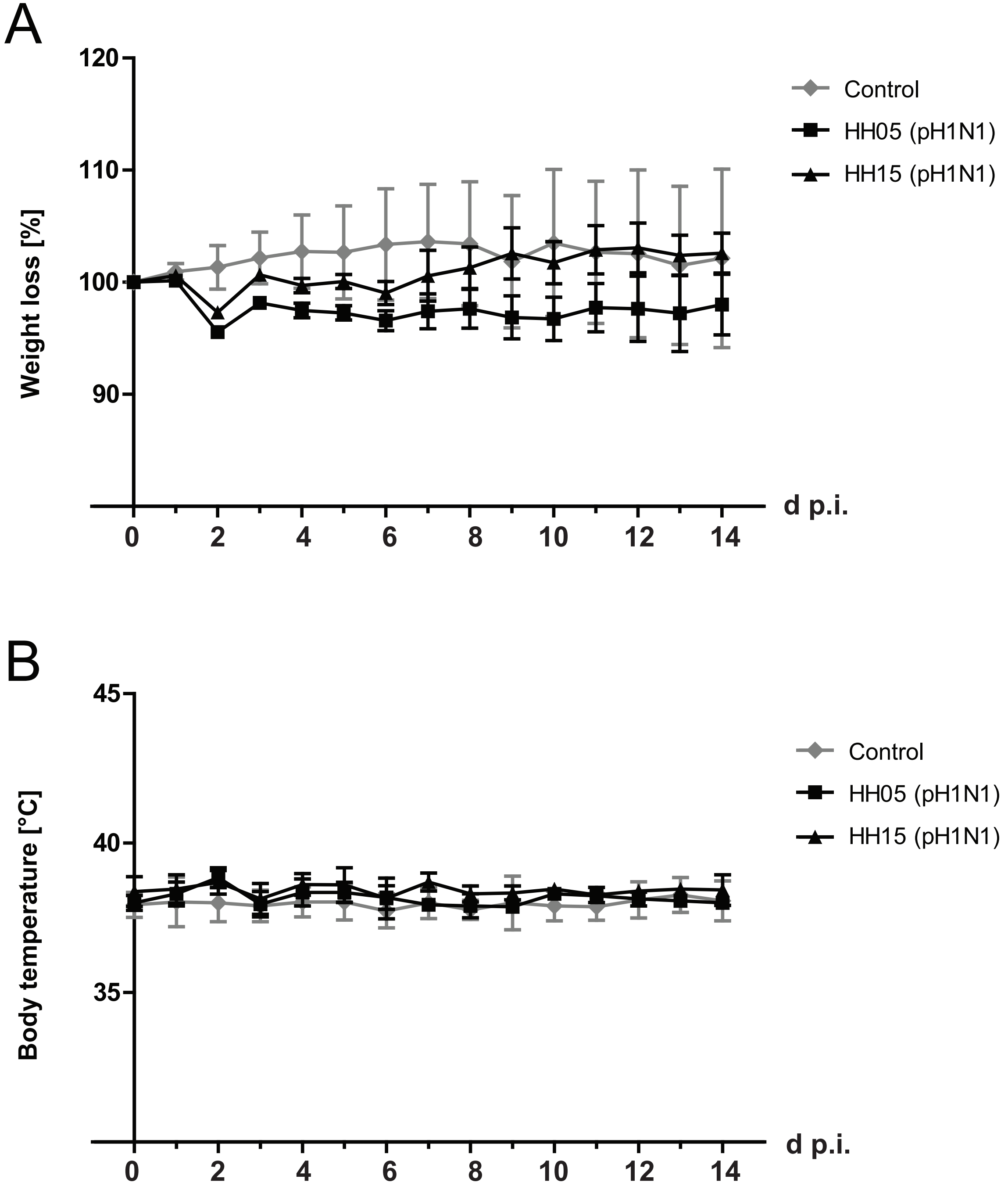

Supplementary Figure 2

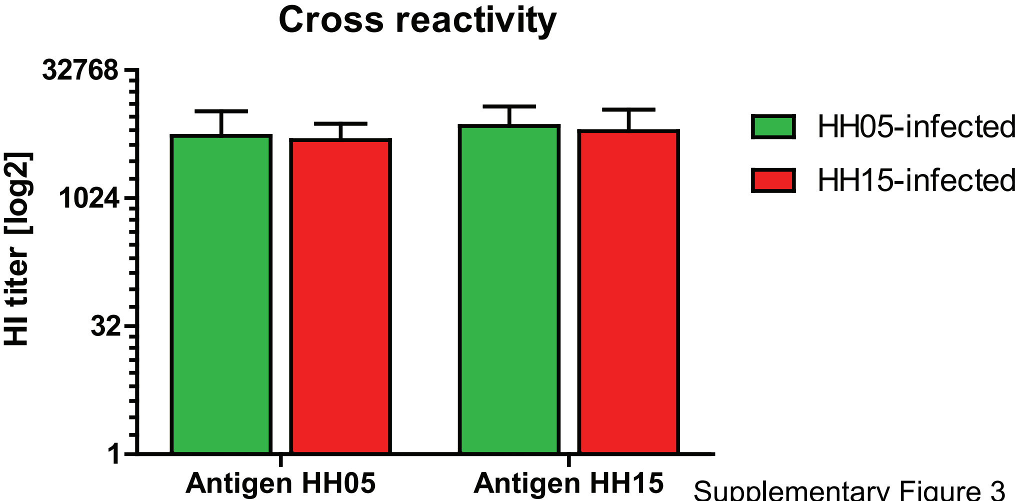

Supplementary Figure 3

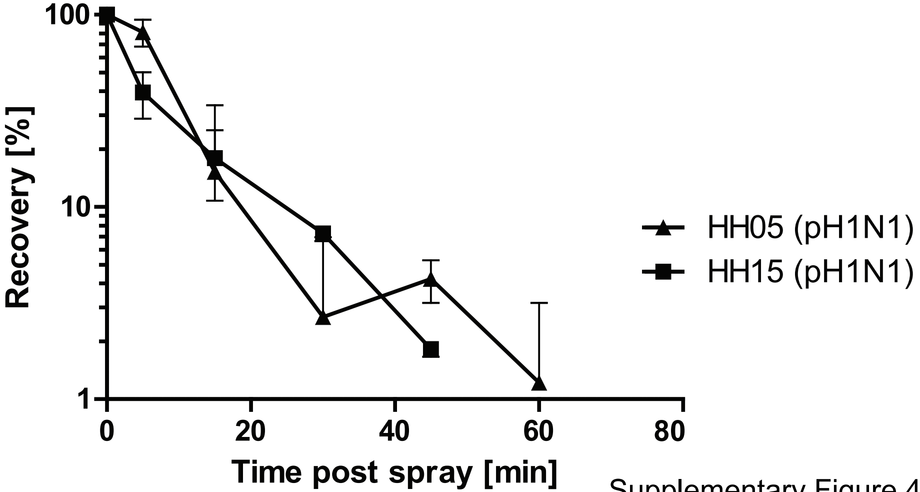

Supplementary Figure 4
